# Proposed treatment strategy for reactive hypoglycaemia

**DOI:** 10.3389/fendo.2024.1332702

**Published:** 2024-02-02

**Authors:** Younes R. Younes, Nicholas Cron, Benjamin C.T. Field, Vidhu Nayyar, James Clark, Sunil Zachariah, Kavitha Lakshmipathy, Jimboy O. Isuga, Negar Maghsoodi, Julian Emmanuel

**Affiliations:** ^1^ Department of Diabetes & Endocrinology, East Surrey Hospital, Surrey & Sussex Healthcare NHS Trust, Redhill, United Kingdom; ^2^ Department of Statistics, London School of Economics, London, United Kingdom; ^3^ Section of Clinical Medicine, Faculty of Health & Medical Sciences, University of Surrey, Guildford, United Kingdom; ^4^ Chemical Pathology Department, University Hospitals Sussex NHS Foundation Trust, Royal Sussex County Hospital, Brighton, United Kingdom

**Keywords:** reactive hypoglycaemia, metformin, mixed meal tolerance test, GLP-1 analogues, freeStyle libre

## Abstract

**Background/aim:**

Managing reactive hypoglycaemia (RH) poses challenges due to limited and often ineffective treatment options. We report a case series and draw on this to propose a stepwise treatment approach consisting of lifestyle modifications, metformin, GLP-1 analogues, and the use of flash glucose monitoring technology.

**Method:**

A retrospective review was conducted to analyse the management of 11 cases presenting with recurrent RH symptoms.

**Result:**

Two patients experienced successful resolution of symptoms through lifestyle modifications. Metformin alone was effective in treating seven out of nine patients who received pharmacological treatment. Two patients with previous upper gastrointestinal surgery showed a partial response to metformin and benefited further from additional long-acting GLP-1 analogue. Pharmacological intervention led to significant reductions in insulin and C-peptide levels in repeat mixed meal tolerance tests (P-values 0.043 for insulin and 0.006 for C-peptide). Finally, flash glucose monitoring technology was useful in early detection and preventing episodes of hypoglycaemia in one of these patients with persistent symptoms.

**Conclusion:**

These findings highlight the potential efficacy of escalated treatment strategies for RH, including the use of metformin, GLP-1 analogues, and flash glucose monitoring technology.

## Introduction

Reactive hypoglycaemia (RH) is a condition characterized by recurrent episodes of hypoglycaemia occurring after consumption of carbohydrate-containing meals (1). Symptoms of RH can vary from mild and self-resolving to severe manifestations such as loss of consciousness, seizures, falls, and impaired cognition that may lead to frequent hospital admissions.

RH is an increasingly recognized and disabling complication of upper gastrointestinal (GI) surgery affecting 10-30% of patients (2, 3). The condition is believed to result from accelerated gastric emptying causing hyperglycaemic peaks and excessive release of the incretin hormones, GLP-1 and GIP, resulting in hyperinsulinemia (4, 5). Furthermore, RH can occur in patients with insulin resistance and mild type 2 diabetes (6). One of the early changes with the onset of type 2 diabetes is a decrease in first-phase insulin release leading to postprandial hyperglycaemia. This is followed by an exaggerated second phase insulin release and subsequent hypoglycaemia (7). The Whitehall II study further supports these findings, demonstrating that insulin secretion increases three to four years prior to the diagnosis of diabetes as a compensatory response to insulin resistance (8). Another form of RH known as idiopathic or functional hypoglycaemia where the cause remains unclear or unknown (9).

Notwithstanding the high prevalence of RH and potential consequences on affected patients, treatment options are limited and often ineffective (10). Dietary advice with frequent small meals, consuming foods high in fibre, and avoiding high glycaemic index foods are effective in stabilizing postprandial glycaemic excursions but difficult to maintain in the long term (11). Medications commonly used include alpha-glucosidase inhibitors (acarbose), which delay the digestion of ingested carbohydrates (12, 13). Calcium channel blockers and diazoxide reduce glucose-stimulated insulin secretion from the pancreatic β cells (14, 15), and somatostatin analogues inhibit the secretion of GLP-1 hormones and insulin (16). These options have potentially troublesome side effect profiles alongside limited efficacy.

We aimed to review our recent clinical experience in managing RH and to use this to assess the efficacy of our treatment strategy for RH, which addresses the causes of glucose fluctuation and of insulin resistance. We found that lifestyle changes, followed, if necessary, by metformin, to reduce demand for post-meal insulin release, then by a long-acting GLP-1 analogue to suppress exaggerated or fluctuating endogenous GLP-1 and, hence, insulin responses, and, finally, by a FreeStyle Libre-2 sensor with an alarm function to warn of impending hypoglycaemia, were effective. We propose this stepwise approach for managing RH patients, in a reproducible treatment algorithm.

## Methods and analysis

We conducted a retrospective review of patients referred to our outpatient endocrine department presenting with symptoms suggestive of RH between September 2017 and August 2022. We comprehensively reviewed clinical records, including symptom profile, symptom severity, hospital/ED attendance, biochemical findings, and treatments implemented. All patients underwent general biochemistry tests and hormonal assessments, including 9 AM cortisol, thyroid function test, and growth hormone to exclude other causes of hypoglycaemia. None of them was taking medications known to cause hypoglycaemia, such as insulin or sulphonylureas. 72 hour fast was performed for patients who reported fasting symptoms.

All patients underwent a mixed meal tolerance test (MMTT) to confirm the diagnosis of RH. The solution used was Fresubin Energy Fibre (200 ml) which consisted of 11.6 g fat, 36 g carbohydrates, 11.2 gram protein, and various vitamins and minerals. A positive test result was defined as glucose level below 3 mmol/L and exaggerated glycaemic excursion in patients with upper GI surgery (17). Once RH was confirmed, dietary changes were the first step implemented in the treatment. If symptoms persisted, metformin and GLP-1 analogues were considered. Finally, Freestyle Libre sensors were an option if symptoms continued. Assessments were conducted at 3-month intervals to monitor the effectiveness of the treatments and for a subset of patients, the MMTT was repeated if symptoms were ongoing to assess biochemical response to treatment. Statistical analysis was performed using repeated measures ANOVA to compare glucose, insulin, and C-peptide values during MMTT before and after interventions. A p-value of < 0.05 was considered statistically significant.

## Results

### Patient’s characteristics

Eleven cases of RH were identified. The basic characteristics of the patients are given in **Table 1**. Seven were females, four males and the median age was 55 ± 10.7 years. Two patients had Roux-en-Y gastric-bypass bariatric operation, one had Nissen fundoplication for gastro-oesophageal reflux disorder (GORD), and another had total gastrectomy for stomach cancer. HbA_1c_ levels in six patients were in the prediabetic range (by ADA criteria), and two patients had been diagnosed with type 2 diabetes prior to their bariatric surgery, with reversal occurring post-surgery.

**Table 1:** Patient characteristics: (N/A, Not available; FSL2, FreeStyle Libre-2; NF, Nissen Fundoplication; RYGB, Roux-en-Y Gastric Bypass; TG, Total Gastrectomy "+", used; "-", not used).

Cases	Age	Gender	BMI (kg/m^2^)	Baseline HbA1c (mmol/mol)	Previous GI surgery	Frequency of symptoms	Intervention				Post intervention HbA1c (mmol/mol)
							Lifestyle	Metformin	GLP-1	FSL2	
**1**	56	F	26.4	39	NF	Daily	+	+	+	+	35
**2**	61	F	42.4	40	RYGB	Daily	+	+	+	–	37
**3**	39	F	27.7	34	RYGB	Daily	+	+	–	–	34
**4**	70	M	N/A	47	No	Monthly	+	+	–	–	45
**5**	55	M	28.4	41	No	Monthly	+	+	–	–	37
**6**	55	F	23	41	No	Daily	+	+	–	–	43
**7**	41	F	27.8	38	No	Every other day	+	+	–	–	37
**8**	61	F	N/A	34	TG	Daily	+	+	–	–	32
**9**	46	F	22.6	35	No	Every other day	+	+	–	–	34
**10**	42	M	N/A	34	No	Daily	+	–	–	–	34
**11**	47	M	N/A	41	No	Monthly	+	–	–	–	N/A

### Clinical presentations of studied participants

All patients reported symptomatic RH episodes within the first few hours of a meal, (typically carbohydrate-laden), that improved with further carbohydrate intake. Case 1, who had previously undergone three fundoplication surgeries for GORD, was referred with frequent daily symptoms of confusion and presyncope, with documented capillary blood glucose falling below 3 mmol/L. Case 2 and 3 both underwent bariatric surgery and within three years began experiencing daily postprandial symptoms of light-headedness, dizziness, sweating, and cognitive impairments. Case 3 symptoms culminated in two seizures requiring admission to ED, both occurring with blood glucose levels <2 mmol/L. Notably, cases 1 and 3 underwent 72 hours fast, and an insulinoma was excluded. Furthermore, they had a trial of diazoxide/acarbose, which was not tolerated. Case 4 experienced two syncopal episodes, and case 5 had three TIA-like instances, within a period of two years, both of which were extensively investigated before hypoglycaemia was suspected, and they were referred for endocrinological evaluations. The remainder of patients had milder, but frequent postprandial symptoms, significantly affecting their daily living, leading to frequent hospital visits and investigations.

### Clinical response to the proposed treatment plan

In the management of all patients, the initial step involved dietary modifications. This included increased frequency of meals (4-5 per day), with a mixed meal composition (nuts, seeds, vegetables, and proteins alongside carbohydrates), and avoiding carbohydrate-laden snacks alone at all times. Cases 10 and 11 responded promptly, their RH symptoms resolved, and their progress was monitored in subsequent follow-ups. The remainder of patients remained symptomatic so pharmacological treatment was instituted.

Cases 1 to 3 & 8, who had history of upper GI surgery (**Table 1**), were started on metformin at 500 mg, gradually increased to 2 g daily, based on their response. Case 3 and 8 reported symptom improvement over an average follow-up duration of 26.5 months. However, for cases 1 and 2, despite metformin improving the frequency and intensity of their RH symptoms, intermittent symptoms persisted. Therefore, as the next step in their therapy, a long acting GLP-1 analogue (dulaglutide) was started at 0.75 mg a week and titrated up to 1.5 mg a week. Case 2’s symptoms markedly improved with the combination therapy over the next 36.5 months of follow-up, while in Case 1, milder symptoms persisted, and GI side effects of therapy were prominent, limiting dose titration. Consequently, we offered her a flash glucose monitoring sensor (FreeStyle Libre-2) with an alarm function which she reported as very useful in predicting and managing impending hypoglycaemia.

In cases 4 to 7 & 9, without a history of GI surgery treatment primarily consisted of lifestyle interventions and metformin, and the dosage was titrated according to the clinical response to maximum dose of 2 g daily. The medication was well tolerated and all patients reported prompt resolution of their symptoms over a mean duration of 19.8 months of follow-up.

### Impact of treatment on incidence of severe hypoglycaemia

**Figure 1:**
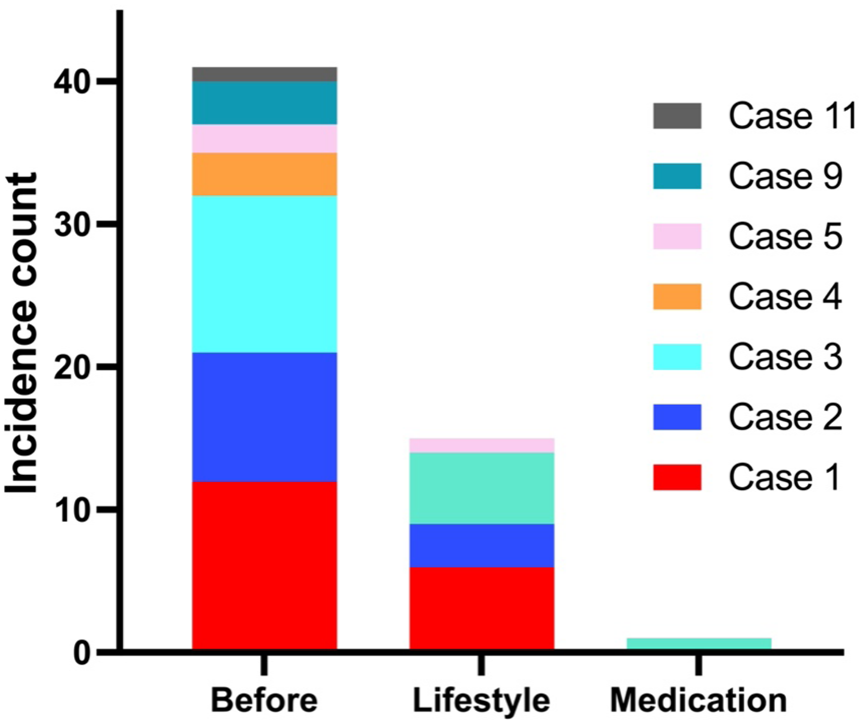
Before intervention, 7/11 patients experienced 41 episodes (32 in cases 1-3 with GI surgery) of severe hypoglycaemia. During lifestyle changes, 4/11 patients had further 15 episodes in a mean follow-up duration of 6.3 (range 3-20) months, and with medications, only 1 episode occurred within 32.4 (16–46) months of follow-up.

We documented the incidence of severe RH in all patients both before and after the interventions (**Figure 1**). Severe hypoglycaemia was defined as episodes requiring assistance from a third party to treat or resulting in hospitalization. Before intervention, 7 out of 11 patients collectively experienced a total of 41 episodes of severe hypoglycaemia, of which 32 episodes occurred in cases 1-3, who had previous GI surgery. Following implementation of lifestyle modifications, an additional 15 episodes (14 in cases 1-3) occurred over a mean period of 6.3 months, while during the pharmacological treatment phase, only one further episode was reported over 32.4 months follow up period. This incident was precipitated by a snack rich in carbohydrates following a long day at work.

### Biochemical response to proposed treatment plan

**Figure 2:**
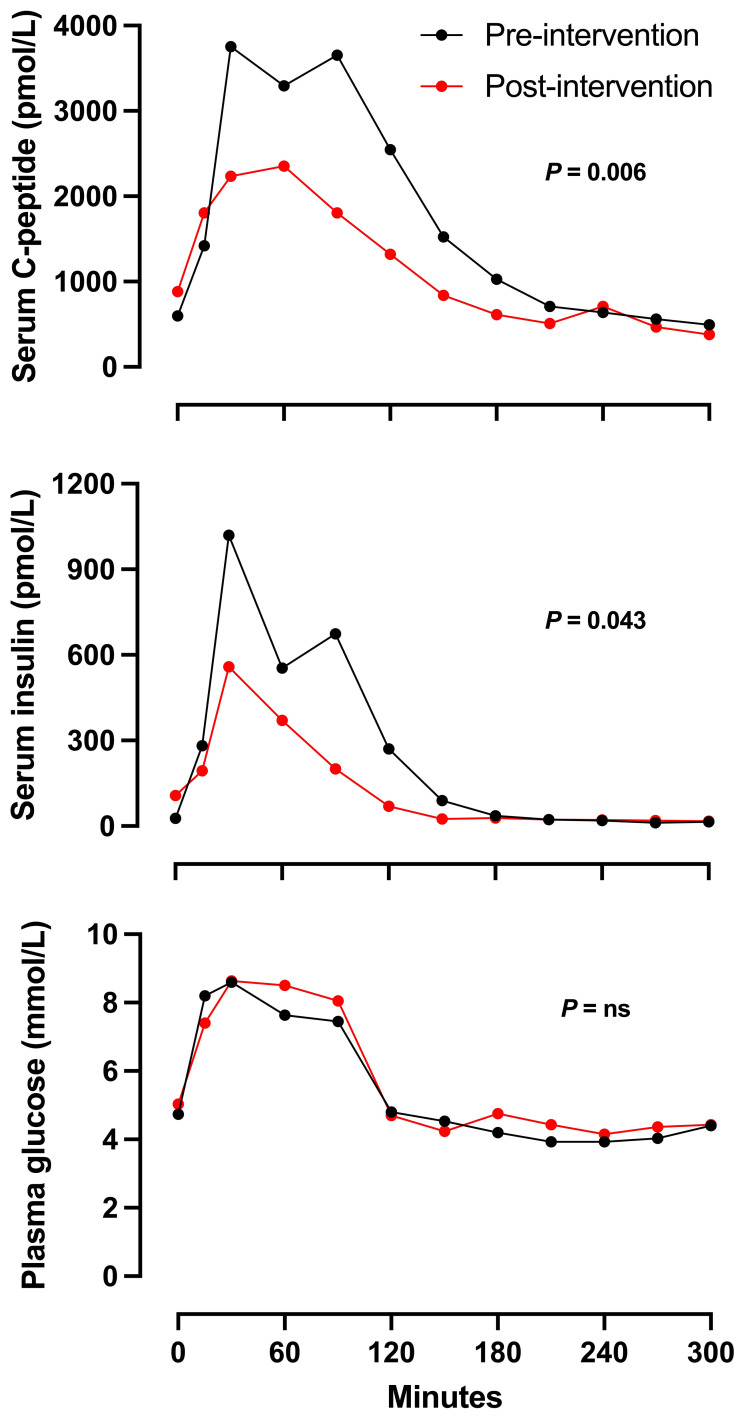
Mean serum C-peptide, serum insulin and plasma glucose concentrations in response to MMTT (200 ml of Fresubin Energy Fibre) in *n* = 3 patients with RH before (Black line) and after intervention (red line). Two patients were treated with lifestyle, metformin and dulaglutide, and one with lifestyle and metformin. Error measures were not included due to small number of patients.

Approximately six months after pharmacological intervention, Cases 1 to 3 underwent a follow-up MMTT to evaluate the impact of the treatment. Although glucose profiles did not change significantly, insulin and C-peptide concentrations were reduced significantly after intervention (P-values 0.043 for insulin and 0.006 for C-peptide), particularly during intermediate time points (30 to 150 minutes; **Figure 2**). These differences suggest a substantial reduction in insulin secretion due to the intervention.

The mean change in HbA1c for patients received metformin was -1 mmol/mol, while for patients on combination therapy, it was -3.5 mmol/mol. (No test for statistical significance was performed).

## Discussion

Our case series confirms that, for patients with RH who fail to respond to lifestyle interventions, a stepwise plan comprising metformin, GLP-1 analogues, and a flash glucose monitoring system can be an effective treatment strategy.

In our case series, metformin alone was effective in treating 7 out of 9 patients who received pharmacological treatment. To our knowledge, notwithstanding the fact that several antidiabetic medications are commonly used “off-label” for RH, this is the first published case series describing the efficacy of metformin in RH. Metformin may be effective via several mechanisms, including inhibiting gluconeogenesis and hepatic glucose output, and improving glucose uptake in muscle, liver, and adipocytes. These actions may lower postprandial blood glucose excursion leading to a reduced tendency to hyperinsulinaemia. Furthermore, metformin may reduce insulin requirements through improved insulin sensitivity (18) and can enhance first phase insulin release (19) which could in turn reduce the requirement for second phase release. Metformin has also shown to slow gastric emptying (20), inhibit rate of intestinal glucose absorption (21), and directly impact islet cells to decrease insulin secretion (22).

Two patients with a history of upper GI surgery who responded only partially to metformin therapy were successfully supplemented with a long-acting GLP-1 analogue. Our case series findings support previous studies that reported successful management of RH with GLP-1 analogues in post-bariatric patients (23–25). It is believed that GLP-1 analogues effectively slow down gastric emptying (26), small intestinal transit (27), decrease postprandial hyperglycaemia, and regulate endogenous GLP-1 excursions, and incretin-stimulated insulin secretion (24). Perhaps weekly GLP-1 analogues, such as Dulaglutide, hold promise in eliminating the daily fluctuations in GLP-1 concentrations after such surgery.

In addition to the clinical benefits observed in our case series with pharmacological treatment, we observed a reduction in C-peptide and insulin levels in repeat MMTTs in three patients. This reduction can be interpreted as evidence supporting the effectiveness of our approach in modulating the hyperinsulinaemia responsible for RH. MMTTs were only repeated if symptoms were ongoing, and the results were used clinically, as a means of illustrating to patients the cause of their symptoms, and the rationale for providing lifestyle modification advice that contradicts their experience of using carbohydrates to terminate symptoms.

Finally, intermittent (“flash”) or continuous glucose monitoring technology, usually used in the management of diabetes mellitus, can also be helpful in RH management. This technology has established itself in many countries as a standard tool for monitoring glucose in insulin-treated patients with diabetes. Symptoms resulting from exaggerated endogenous insulin secretion can also be managed with this technology. We have used glucose sensors for our patients with RH when pharmacological interventions have reached their ceiling effect, although this is currently off-label (28). Their use can alert patients of impending hypoglycaemia, so they can take evasive action. However, it is crucial to inform patients about potential drawbacks, as using this technology might lead to increased snacking when glucose downtrend. Hence, patient education remains crucial. In our patient, this has not led to weight fluctuations.

We acknowledge limitations to our conclusions resulting from the case series design. In particular, this constitutes a retrospective analysis of patients presenting with symptoms, without a control group, and the number of patients is relatively small, although RH causing severe hypoglycaemic episodes is not a common endocrine pathology. Furthermore, we have assessed efficacy by recording patients’ reports of the effect of treatment on hypoglycaemic symptoms and on severe hypoglycaemia incidence, on the assumption that resolution of symptoms demonstrates cure. In patients for whom glucose monitoring data were not available, we cannot exclude the possibility that asymptomatic hypoglycaemia might nevertheless have occurred. Additionally, we undertook repeat MMTT only if further investigation was indicated on clinical grounds. We acknowledge that this limits the scope for assessment of the intervention.

In conclusion, our case series provides evidence supporting the efficacy of a stepwise treatment strategy for patients with RH who do not respond to lifestyle interventions. Metformin and GLP-1 analogues demonstrated positive outcomes in improving symptoms with corresponding reductions in insulin and C-peptide levels. Additionally, the use of intermittent or continuous glucose monitoring technology is valuable for early detection and prevention of hypoglycaemic episodes. These findings highlight the potential of this treatment approach in the management of RH but a prospective study is needed to validate its efficacy.

## Data availability statement

The original contributions presented in the study are included in the article/supplementary material. Further inquiries can be directed to the corresponding author.

## Ethics statement

Ethical approval was not required because the use of metformin is in line with currently accepted UK clinical practice for the management of postprandial hypoglycaemia in the context of insulin resistance. Other medications commonly used in this setting include alphaglucosidase inhibitors, DPP4 inhibitors, GLP-1 receptor agonists, somatostatin analogues and diazoxide. Our manuscript reports a service evaluation, in the form of a case series, that is entirely retrospective and observational in nature, drawing only on routinely collected clinical data. As such, no ethical approval was required.

## Author contributions

YY: Methodology, Writing – original draft, Writing – review & editing. NC: Formal Analysis, Writing – original draft. BF: Conceptualization, Formal Analysis, Supervision, Writing – review & editing. VN: Conceptualization, Writing – review & editing. JC: Conceptualization, Writing – review & editing. SZ: Conceptualization, Writing – review & editing. KL: Conceptualization, Writing – review & editing. JI: Conceptualization, Writing – review & editing. NM: Conceptualization, Writing – review & editing. JE: Conceptualization, Formal Analysis, Methodology, Supervision, Writing – original draft, Writing – review & editing.
